# Neurological complications during the Omicron COVID‐19 wave in China: A cohort study

**DOI:** 10.1111/ene.16096

**Published:** 2023-10-25

**Authors:** Lu Lu, Lei Chen, Peiyu Wang, Zhang Qi, Yujie Chen, Xintong Wu, Xu Liu, Minjin Wang, Jinmei Li, Bo Yan, Jian Guo, Sisi Teng, Weimin Li, Josemir W. Sander, Dong Zhou, Weixi Xiong

**Affiliations:** ^1^ Department of Neurology West China Hospital of Sichuan University Chengdu Sichuan China; ^2^ Institute of Brain Science and Brain‐inspired Technology of West China Hospital Sichuan University Chengdu Sichuan China; ^3^ Department of Neurology Chengdu ShangJin NanFu Hospital Chengdu Sichuan China; ^4^ Department of Neurology West China Tianfu Hospital Chengdu Sichuan China; ^5^ Department of Pulmonary and Critical Care Medicine, West China Hospital Sichuan University Chengdu Sichuan China; ^6^ Tianfu Jincheng Laboratory Chengdu Frontier Medical Center Chengdu Sichuan China; ^7^ UCL Queen Square Institute of Neurology London UK; ^8^ Chalfont Centre for Epilepsy Chalfont St Peter UK; ^9^ Stichting Epilepsie Instellingen Nederland (SEIN) Heemstede The Netherlands

**Keywords:** encephalitis, inpatient, SARS‐CoV‐2

## Abstract

**Background and purpose:**

The aim was to investigate the neurological complications associated with coronavirus disease 19 (COVID‐19) during the 2022 Omicron wave.

**Methods and analysis:**

The medical records of a cohort of people admitted to neurological wards of three participating tertiary centres in Sichuan from 12 December 2022 to 12 January 2023 were reviewed. Demographics and clinical data were obtained and analysed with an interest in COVID‐19‐related new‐onset or worse neurological symptoms. The current data were also compared in two centres with similar data from the same period 12 months earlier.

**Results:**

In all, 790 people were enrolled, of whom 436 were positive for COVID‐19. Ninety‐nine had new onset COVID‐related neurological problems, or their known neurological condition deteriorated during the wave. There was a significant difference in demographics from the findings amongst admissions 12 months earlier as there was an increase in the average age, the incidence of encephalitis and encephalopathy, and mortality rates. One hundred and one received COVID‐specific antivirals, intravenous glucocorticoids and intravenous immunoglobulin therapy. No differences were seen between these and those who did not use them.

**Conclusion:**

New‐onset neurological conditions, particularly encephalitis and encephalopathy, increased significantly during this period. Deterioration of existing neurological conditions, such as seizure exacerbation, was also observed. A large‐scale treatment trial of people with COVID‐19 infection presenting with neurological disorders is still needed.

## INTRODUCTION

Coronavirus disease 2019 (COVID‐19), caused by severe acute respiratory syndrome coronavirus 2 (SARS‐CoV‐2) infection, has resulted in a global pandemic. Many strains have emerged since the original wave, including the Omicron variant. It was first reported in South Africa in November 2021. Omicron was found to be highly transmissible due to mutations on the spike protein [1], but most affected people only had mild symptoms, not requiring hospital admission. In 2020, it was reported that the frequency of new‐onset critical neurological events was 3.5% [2]; however, whether Omicron will cause other neurological involvements remains unknown. Since December 2022, China has faced widespread COVID infection. According to the Chinese Center for Disease Control data, the Omicron subvariants BA.5.2 and BF.7 predominantly led this [3]. The vaccination peak was a year before this wave, so the Omicron‐specific neutralizing antibodies were probably relatively low [4]. Many cases quickly emerged in China after relaxation of the COVID rules, as most people had no prior exposure to any SARS‐CoV‐2 strains and most people had non‐mRNA COVID vaccines [5]. Admission to neurological wards was assessed during the Omicron waves and was compared to similar data from 12 months earlier.

## METHODS

### Ethics approval

The West China Hospital Ethics Committee (2023 [30]) approved the study. All participants provided written consent.

### Patient and public involvement

Due to the acute situation, no public or patient involvement in the study design was possible.

### Study population

This cross‐section study was conducted in three tertiary hospitals in Chengdu, Sichuan (West China Hospital of Sichuan University, Chengdu ShangJin NanFu Hospital and West China Tianfu Hospital). People discharged from neurology wards were consecutively enrolled from 12 December 2022 to 12 January 2023. Those admitted before 12 December 2022 were excluded as this was taken as the onset of this specific COVID‐19 wave.

As the control cohort, people discharged from the neurology wards in the same period 1 year earlier were used. This was during the period in which the whole population in the area was regularly tested for COVID‐19, and admission to the hospitals was conditional on three negative polymerase chain reaction (PCR) tests. Tianfu Hospital started to admit neurological cases on 18 February 2022. Therefore, year‐on‐year comparative data were not available for admission to this hospital.

### Data acquisition

A standardized form was designed to extract clinical features, test results, COVID infection history and medical history data. Seven neurologists completed the form through an online platform (https://www.wjx.cn/vm/wcGF5fg.aspx). Clinical information was extracted from electronic medical records and telephone follow‐ups. In the case of uncertainty, attending physicians or neurologists were contacted.

The diagnosis of the SARS‐CoV‐2 infection was made according to the 9th version of the Chinese national COVID‐19 treatment guideline. Those included were further stratified as having mild, moderate, severe or critical conditions based on the above guideline. A bi‐stratification into critical (severe and critical levels) and noncritical (mild and moderate) is used in the statistical analysis of different neurological manifestations.

### Definitions

A COVID‐related admission was defined as new‐onset neurological symptoms or signs timely related to the individual's COVID infection history. The admission reasons were classified as COVID‐related new‐onset neurological manifestation, COVID‐related deterioration of the existing neurological disease, unrelated neurological manifestation, neurological manifestation with an unclear relationship with COVID, and respiratory or systemic disorders. People who developed new‐onset neurological symptoms after or during COVID infection (If the time interval between new neurological symptom and Covid‐19 symptoms >3 days) but could not find the clear time−symptom link were assigned to the unclear group. A PCR or antigen test result was necessary to support a COVID infection.

### Real‐time PCR test in cerebrospinal fluid

Amongst individuals with probable COVID‐19‐related encephalitis or encephalopathy, who had an indication for lumbar puncture, an approach was used to test the presence of SARS‐CoV‐2 in their cerebrospinal fluid (CSF). After a complete clinical‐analytical CSF examination, every other sample was tested based on a random approach due to the test capacity, if there was still CSF leftover.

Real‐time PCR was performed by amplifying two target genes, open reading frame 1ab (ORF1ab) and nucleocapsid protein (N), using a qRT‐PCR kit (Sansure Biotech Inc.) with a real‐time PCR thermal cycler (ABI 7500 system, Applied Biosystems). Each test had to be repeated, and the results had to be consistent twice before they were accepted and recorded.

### Statistical analysis

The ANOVA test and chi‐squared test were applied based on the variables. IBM SPSS Statistics was used for the analysis. Results were considered statistically significant at a *p* value of 0.05.

### Baseline characteristics

**FIGURE 1:**
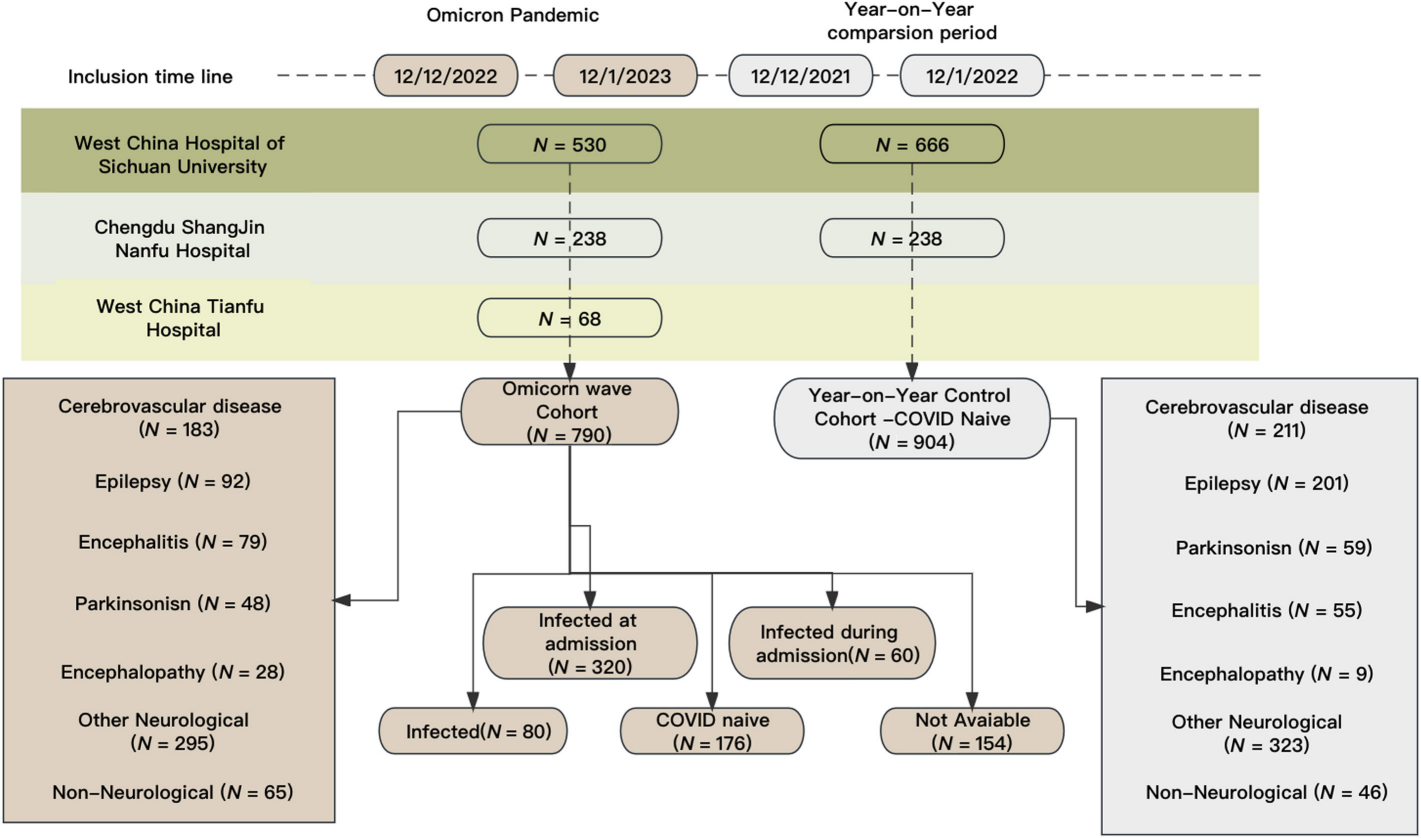
Flowchart of study enrolment.

In all, 790 inpatients were enrolled: 530 from West China Hospital, 192 from ShangJin NanFu Hospital and 68 from Tianfu Hospital. Fifty‐five per cent (*N* = 436) tested positive at the admission screening. COVID‐19 symptoms in 144 (33%) were classified as mild, 234 (54%) as moderate, 33 (8%) as severe and 25 (6%) as critical. None of the participants reported previous COVID‐19 infection before this wave (Figure 1). The baseline characteristics of the whole cohort are provided in Table 1. There is no significant difference in age, gender, COVID infection history and length of stay.

**TABLE 1:** The baseline characteristics in three centres.

	West China Hospital	ShangJin NanFu Hospital	Tianfu Hospital	Total	*p*
Number	530	192	68	790	
Male (*N*, %)	301 (56.8)	103 (53.6)	32 (47.1)	436 (55.2)	0.279
Age (years, mean ± SD)	53.5 ± 21.8	55.2 ± 20.6	54.5 ± 22.5	54.0 ± 21.5	0.61
Route of admission					
Outpatient clinic	266 (49.8)	133 (30.7)	38 (44.1)	437 (44.7)	*p* < 0.00
Emergency	264 (50.2)	59 (69.3)	30 (55.9)	351 (55.3)	
SARS‐CoV‐2 PCR test results at admission					
Positive	211 (39.8)	90 (46.9)	23 (33.8)	324 (41.0)	0.051
Negative	230 (43.4)	62 (32.3)	20 (29.4)	312 (39.5)	
NA	89 (16.8)	40 (20.8)	25 (36.8)	154 (19.5)	
COVID infection history					
Positive at admission	211 (47.8)	86 (56.6)	23 (53.5)	320 (50.3)	0.094
Infected during admission	41 (9.3)	18 (11.8)	1 (2.3)	60 (9.4)	
Known to be positive before admission	57 (12.9)	19 (12.5)	4 (9.3)	80 (12.6)	
COVID naive	132 (29.9)	29 (19.1)	15 (34.9)	176 (27.7)	
Length of admission (days, mean ± SD)	7.4 ± 4.8	8.2 ± 5.4	8.2 ± 6.6	7.6 ± 5.2	0.127
In‐hospital mortality (*N*, %)	28 (5.3)	3 (1.6)	28 (5.3)	3.5%	0.033

Abbreviations: COVID, coronavirus disease 19; PCR, polymerase chain reaction; SARS‐CoV‐2, severe acute respiratory syndrome coronavirus 2.

Seventy per cent of the COVID‐positive individuals had a computed tomography chest scan, which suggested viral pneumonia in 54%. The classification significantly affected the length of stay (*p* = 0.002). The core neurological manifestations in these individuals combined with COVID infection are shown in Table 2.

**TABLE 2:** Core neurological manifestations in people with critical infection of COVID.

	Critical infection (*N*, %)	Non‐critical infection (*N*, %)	*p*
Cerebrovascular disease	15 (12.8)	102 (87.18)	0.857
Epilepsy	2 (8.0)	23 (92.0)	0.616
Encephalitis	9 (15.0)	51 (85.0)	0.677
Encephalopathy	8 (30.77)	18 (69.23)	0.007
Parkinsonism	3 (10.3)	26 (89.7)	0.84
Demyelination	0	21 (100)	0.184
Other neurological manifestations	7 (6.4)	103 (93.6)	0.013
No specific neurological manifestations	14 (29.17)	34 (70.8)	0.382
Total	58 (13.3)	378 (86.7)	

Abbreviation: COVID, coronavirus disease 19.

The reasons for admission were classified into five categories.

- Forty‐four individuals (5.6%) had new‐onset neurological symptoms or signs related to COVID. The most frequently reported symptoms included altered consciousness, such as delirium or decreased consciousness level, seizures and focal limb weakness.
- Fifty‐five people (7%) with pre‐existing neurological conditions (including Alzheimer's disease, stroke, Parkinson's disease, amyotrophic lateral sclerosis, migraine, myasthenia gravis, demyelination disease like neuromyelitis optica spectrum disorders, chronic inflammatory demyelinating polyneuropathy and multiple sclerosis) presented with a significant deterioration of their clinical pictures concomitant with COVID symptoms. Eleven individuals with epilepsy were amongst them, with seizure recrudescence or status epilepticus coinciding with the infection. Individuals have also been seen with autoimmune peripheral nerve disorder who paused glucocorticoid therapy during the infection with subsequent deterioration.
- A total of 174 individuals (22%) had neurological manifestations with an unclear relationship to COVID infection. They had tested positive at the onset of their acute stroke (70) and acute encephalitis or encephalopathy (43, one with recreational drug use) and other acute neurological complaints, which included vision impairment, myelitis, seizures, severe headaches, dizziness and worsening in their well‐controlled neurological disease, including neuromyelitis optica, subacute combined degeneration, myasthenia gravis. The onset of the neurological exacerbation was challenging to define, but all had tested positive at admission.
- There were 458 admissions (58.0%) for unrelated neurological diseases. The symptom onset of this group dates back to December 2022, when none of them was positive, according to the governmental database.
- Some individuals with respiratory or systemic complaints without a neurological history were admitted to the wards during the period for logistical reasons.

### Year‐on‐year comparison

In all, 666 admissions from West China Hospital and 238 from ShangJin NanFu Hospital were used as the year‐on‐year control group. All 904 individuals from the previous year had negative SARS‐CoV‐2 PCR results on admission and reported no COVID‐19 infection during hospitalization. The clinical characteristics of the two groups are shown in Table 3. Thirty‐one deaths during admission were reported; the deceased's clinical features are shown in Table 4.

**TABLE 3:** Year‐on‐year comparison.

	2022	2023	*p*
Number	904	722	
Male (*N*, %)	485 (53.7)	404 (56.0)	0.354
Age (years, mean ± SD)	50.17 ± 20.00	53.94 ± 21.47	*p* < 0.00
Route of admission			
Outpatient clinic	673 (74.4)	399 (55.3)	*p* < 0.00
Emergency	231 (25.6)	323 (44.7)	
Length of admission (days, mean ± SD)	6.53 ± 4.30	7.58 ± 5.01	*p* < 0.00
In‐hospital mortality (*N*, %)	4 (0.4)	33 (4.6)	*p* < 0.00
Interval between symptoms onset (*N*, %)			
New‐onset neurological symptoms within 7 days	139 (15.4)	169 (23.4)	*p* < 0.00
Worse within 7 days	35 (3.9)	54 (7.5)	
Chronic neurological disease	682(75.4)	443(61.4)	
Non‐neurological disease	48 (5.3)	56 (7.8)	
Ratio of primary diagnosis (*N*, %)			
Cerebrovascular disease	211 (23.3)	183 (25.3)	0.348
Epilepsy	201 (22.2)	92 (12.7)	*p* < 0.00
Encephalitis	55 (6.1)	79 (10.9)	*p* < 0.00
Parkinsonism	59 (6.5)	48 (6.6)	0.922
Other movement disorder	25 (2.8)	4 (0.6)	0.002
Dementia	23 (2.5)	10 (1.4)	0.1
Demyelination	36 (4.0)	34 (4.7)	0.473
Encephalopathy	9 (1.0)	28 (3.9)	*p* < 0.00
Neuromuscular junction disease	26 (2.9)	24 (3.3)	0.603
Peripheral neuropathy	38 (4.2)	23 (3.2)	0.283
Dizziness	25 (2.8)	25 (3.5)	0.419
Headache	24 (2.7)	24 (3.3)	0.428
Other neurological disease	111(12.3)	68 (9.4)	0.067
Non‐neurological disease	46 (5.1)	65 (9.0)	0.002

**TABLE 4:** Characteristics of in‐hospital deaths.

Number	Throat swab PCR at admission	COVID‐19 classification	Cause of death	Core neurological diagnosis
1	Negative	Critical	Respiratory circulatory failure	Acute cerebral infarction
2	Positive	Critical	Respiratory circulatory failure	Acute cerebral infarction
3	Positive	Critical	Circulatory failure	Acute cerebral infarction
4	Positive	Critical	Septic shock	Acute cerebral infarction
5	Positive	Critical	Severe pneumonia	Acute cerebral infarction
6	Positive	Critical	Severe pneumonia	Acute cerebral infarction
7	Negative	Never infected	Cerebral herniation	Acute cerebral infarction
8	Negative	Severe	Respiratory circulatory failure	Acute cerebral infarction with haemorrhage transformation
9	Negative	Never infected	Respiratory circulatory failure	Acute cerebral infarction with haemorrhage transformation
10	Positive	Critical	Severe pneumonia	Cerebrovascular disease
11	Negative	Moderate	Respiratory circulatory failure	Cerebrovascular disease
12	Positive	Critical	Respiratory circulatory failure	Convulsive status epilepticus
13	Positive	Critical	Multiple organ dysfunction syndrome	Cryptococcal meningoencephalitis
14	Positive	Critical	Respiratory failure	Encephalitis
15	Negative	Critical	Respiratory circulatory failure	Encephalitis
16	Negative	Mild	Severe pneumonia	Encephalitis
17	Positive	Moderate	Cerebral herniation	Intracranial haemorrhage
18	Positive	Moderate	Cerebral herniation	Intracranial infection
19	Positive	Severe	Respiratory circulatory failure	Intracranial infection
20	Positive	Mild	Cerebral herniation	Intracranial infection
21	Positive	Critical	Tuberculous meningitis	Tuberculous meningitis
22	NA	Never infected	Respiratory circulatory failure	Neuromyelitis optica spectrum disorders
23	Positive	Mild	High falls	Psychobehavioural abnormality
24	Positive	Critical	Respiratory circulatory failure	Toxic encephalopathy
25	Positive	Critical	Toxic encephalopathy	Toxic encephalopathy
26	Positive	Critical	Septic shock	Metabolic encephalopathy
27	Positive	Critical	Respiratory circulatory failure	Metabolic encephalopathy
28	Positive	Critical	Cerebral herniation	Intracranial metastases
29	NA	NA	Intracranial metastases	Intracranial metastases
30	Positive	Critical	Cerebral herniation	Intracranial metastases
31	Negative	Never infected	Severe pneumonia	NA

Abbreviations: COVID, coronavirus disease 19; PCR, polymerase chain reaction.

Compared to the previous year, the total number of admissions decreased by a quarter, with a significant change in pathologies and reasons for admission. An increase in cases of encephalitis and encephalopathy was seen, whilst a substantial decrease in selective admissions for epilepsy (mainly for video electroencephalograph recording), parkinsonism and other movement disorders, and dementia was seen. The considerable difference in the non‐neurological disease ratio in the 2 years reflected a disease shift in this group from somatoform disorder and sleep disorder to respiratory illness.

Whilst the total cerebrovascular disease ratio still steadies this year, the National Institutes of Health Stroke Scale (NIHSS) in acute stroke at admission showed no significant difference (5.42 ± 6.32 vs. 5.49 ± 7.46).

### Findings in cerebrospinal fluid

The significant rise in encephalitis and encephalopathy admissions has driven attention to CSF. Eighteen spare CSF tests were allocated. These were used amongst those suspected of probable COVID‐19 encephalitis or encephalopathy requiring lumbar puncture. Real‐time PCR tests were used in nine samples. All nine were tested for CSF PCR, and their throat swab PCR was positive when admitted. Four people had positive results, including three new‐onset encephalitides with unknown aetiology and one metabolic encephalopathy. Antiviral treatments were used in two and intravenous immunoglobulin in three. The cases that only used antiviral therapy showed minor clinical improvement. The other five CSF examinations from new‐onset encephalitis were negative. One of the five was later diagnosed with glial fibrillary acidic protein astrocytopathy.

### Treatment‐specific analysis

Amongst the 436 individuals with in‐hospital COVID infection, 22 were treated with Paxlovid and 25 with Azvudine. Three were treated with both, including two with critical COVID illness and one with moderate disease and new‐onset encephalitis. Using Paxlovid significantly increased the length of stay (8.57 ± 5.11 vs. 12.41 ± 5.41, *p* = 0.001) but showed no difference in mortality (*p* = 0.272). Conversely, using Azvudine did not impact the length of stay or mortality. No acute neurological treatment emerged in these individuals during the application of these drugs.

Sixty‐two people used glucocorticoids during the course, and another 29 were given immunoglobulins and they had a significantly longer length of stay.

## DISCUSSION

In this multi‐centre prospective cohort study, people admitted to neurology wards were enrolled, and most had COVID infection during the wave. Compared to data from the previous year, age, disease distribution, admission route, rate of onset of neurological symptoms, length of admission and in‐hospital mortality were significantly changed. Although most people only had mild or moderate symptoms of COVID, one‐third had new‐onset neurological symptoms or worsening of pre‐existing neurological disease. No severe adverse side effects of Paxlovid or Azvudine were seen.

Reports of neurological manifestations of Omicron infection were initially rare. In one study of non‐severe cases of Omicron infection, a few cases of acute cerebrovascular disease, impaired consciousness and seizure were reported. Others were non‐specific neurological symptoms that were highly subjective and unspecific [6]. Our pre‐Omicron report showed a very low rate of neurological complications during the infection. Similar studies had a frequency of neurological complications ranging from about a third to about two‐thirds, mostly non‐specific symptoms [7, 8, 9].

With China's previous strict zero‐COVID policy, most people did not get infected with COVID‐19 before this Omicron wave. Unexpectedly, the Omicron spread largely unmitigated across the country, and according to the Chinese Center for Disease Control the massive outbreak peaked around 22 December with 7 million new cases daily [10]. Although most infections were mild and no hospitalization was required, many cases still overwhelmed hospitals. Our data showed a decrease in admission compared to a non‐COVID period, whose causes might be multifactorial, including severe staff shortages and longer length of stay of the COVID‐19 cases.

Multiple studies showed that essential health services for chronic noncommunicable diseases were reduced or disrupted during the COVID‐19 pandemic, potentially leading to a worse outcome or higher risk of death [11, 12]. The comparison shows a dramatic increase in emergency admissions. One of the main reasons was the COVID‐related acute neurological symptoms, as more people had new‐onset or worsened neurological symptoms within 7 days in the COVID wave compared to last year. Compared to the previous year, our cohort also had a higher rate of non‐neurological diseases, mostly pneumonia, as according to national guidelines people with critical illnesses in the emergency department needed to be admitted within 24 h. Likewise, some acute neurological diseases, including cerebrovascular diseases, had similar admissions, but some chronic diseases admitted for evaluation (epilepsy) or treatment (movement disorder) decreased hugely this year.

Similar incidences of stroke were seen in the two time periods. One study reported a higher incidence of stroke in centres focused on neurological conditions rather than general cohorts [13]. Furthermore, the average NIHSS score showed no difference between the two periods, despite a recent review suggesting that stroke severity was higher in people having a stroke in the context of COVID‐19. This may be multifactorial. First, the review only included studies in the pre‐Omicron era; our subjects were infected with Omicron. Secondly, it also reflected the differences in the population assessed. Although people with COVID‐19 were reported to have a characteristic stroke profile, whether the Omicron variant also significantly impacts the cerebrovascular system is unclear.

Encephalitis related to COVID‐19 infection has been broadly reported since the onset of the outbreak. One large international multi‐centre cohort study of inpatients reports a rare incidence of meningitis and/or encephalitis [13], which agrees with the number from a systematic review and meta‐analysis [14]. The review also showed that severely ill people had a higher risk of developing encephalitis. Another large cohort of COVID‐19 individuals with encephalitis showed that most of them only had moderate COVID‐19 regarding respiratory illness, similar to our findings [15]. Multiple cohorts in the pre‐Omicron age reported a high incidence of acute encephalopathy in people infected with COVID‐19 [13, 16, 17]. In one sizeable multi‐centre study, it was the most common neurological complication in all COVID‐19 cases [13]. An increasing trend of encephalopathy was also witnessed in our cohort compared to the data of the same period of last year. People with severe or critical COVID‐19 were more likely to develop encephalopathy than mild cases. In our data, the exact incidence of encephalopathy and encephalitis was not known and was compared with the previous year, which showed a significant increase. A community‐based study of human incidence is warranted.

Establishing a causality link between Omicron and encephalitis was not feasible in our study as no pattern was observed in the clinic manifestation. CSF PCR was unavailable for most cases due to a lack of supply. Severe encephalitis/encephalopathy has been reported frequently in children, including neonates with Omicron infection [18, 19, 20, 21], and fewer cases were reported in adults [22, 23, 24]. Most CSF samples tested negative for the Omicron variant [18, 22, 23, 24]. One study compared the risk of encephalitis/encephalopathy when infected with Omicron with parainfluenza virus and influenza virus; interestingly, the risk in Omicron was between parainfluenza virus and influenza virus [19]. No data on the risk in adults compared to other viral infections are available, so studies on this risk are needed.

A much higher death rate is reported than the data in the same period last year. Most cases had definite COVID infection; in the infected cases, most were severe or critical COVID. Reports mentioned that people having any neurological complaints were more likely to have died [13]. In our cohorts, people with acute cerebral accident and encephalitis comprised the most seen diagnosis in the deceased group. However, most people die of pneumonia or systemic conditions rather than the primary neurological disease, suggesting early antiviral treatment may have a role in reducing mortality.

In our cohort, only a small group received Paxlovid or Azvudine due to a lack of supply, so the data were small to get convincing evidence. Previous studies do not support changing the approach to acute stroke management in people infected with COVID‐19. In people with encephalitis/encephalopathy, antivirals and corticosteroids were used based on experience and without high‐quality evidence [22]. Previous cases also had a successful experience using early combined antiviral therapy or hybrid continuous renal replacement therapy and plasma exchange in critical encephalopathy [24, 25]. Neurological‐specific treatment needs further assessments.

Our study has several limitations. First, our analysis was retrospective; however, multiple centres were included, one of which is the largest hospital in West China, and all participants were enrolled consecutively during the study period. Secondly, care should be exercised when generalizing our findings to other populations. The high incidence or severity of neurological complications could be due to a lack of exposure to any COVID‐19 variants due to the strict social‐distancing practices in China and the lack of efficacy of the Chinese vaccines. Thirdly, the in‐hospital mortality was hugely biased compared with that in the community as many mild cases were not admitted. In the future, follow‐up studies are worthy of further investigation.

## CONCLUSION

Omicron might trigger new‐onset neurological conditions or worsen the pre‐existing neurological disease, and some might lead to severe life‐threatening illness, including encephalitis/encephalopathy. A higher mortality rate and changed disease constituent ratio were seen than in last year's data. Pathogen investigations in the CSF should be considered early to guide the treatment. The booster of COVID‐19 has been confirmed to lower mortality; a larger campaign of vaccination should be proposed to protect vulnerable populations.

## AUTHOR CONTRIBUTIONS

Lu Lu: Methodology; investigation; formal analysis; writing—original draft; writing—review and editing. Lei Chen: Investigation; data curation; validation; funding acquisition; writing—review and editing; formal analysis. Peiyu Wang: Data curation; investigation. Zhang Qi: Investigation; data curation. Yujie Chen: Data curation; investigation. Xintong Wu: Data curation; investigation. Xu Liu: Investigation. Minjin Wang: Investigation; data curation. Jinmei Li: Data curation; supervision; investigation. Bo Yan: Investigation; data curation; supervision. Jian Guo: Data curation; supervision; investigation. Sisi Teng: Data curation. Weimin Li: Conceptualization; investigation; funding acquisition; validation; project administration. Josemir W. Sander: Writing—review and editing; conceptualization; investigation; validation; methodology; formal analysis. Dong Zhou: Conceptualization; funding acquisition; writing—review and editing; methodology; project administration. Weixi Xiong: Conceptualization; methodology; writing—review and editing; funding acquisition; supervision; project administration.

## CONFLICT OF INTEREST STATEMENT

JWS reports personal fees from Eisai, UCB Pharma and Angelini Pharma outside this work. All other authors have no disclosures to make.

## Data Availability

The data that support the findings of this study are available on request from the corresponding author. The data are not publicly available due to privacy or ethical restrictions.
